# Laparoscopic Cholecystectomy for Cholelithiasis in Patients With Liver Cirrhosis

**DOI:** 10.1155/DTE.1.147

**Published:** 1995

**Authors:** Tetsuro Ishikawa, Michio Sowa, Masayoshi Nagayama, Yukio Nishiguchi

**Affiliations:** First Department of Surgery Osaka City University Medical School 1-5-7 Asahi-machi Abeno-ku Osaka 545 Japan

## Abstract

We performed laparoscopic cholecystectomy for symptomatic cholelithiasis on four patients with cirrhosis of the liver, two of whom had clinical portal hypertension and splenomegaly. Preoperative examination disclosed hypersplenism in one patient, while mild thrombocytopenia and decreased prothrombin concentration were noted in three patients. However, no remarkable bleeding tendency was recognized clinically in any of the patients. Preoperatively, by Child-Pugh's criteria, three patients had class B disease and one class A disease. Intraoperatively, remarkable inflammatory change or fibrotic change of the gallbladder wall and Calot's triangle was observed in two cases, and collateral veins and lymphangial congestion were observed in all four cases. In the first case, extreme bleeding and lymphorrhea from dissected sites were observed, and a 1.5 unit of transfusion of whole blood was required during operation. Postoperatively, increase in ascites which was controlled with diuretics was recognized in one case. However, the postoperative course was uneventful in all cases, and no serious complications were recognized.

That laparoscopic cholecystectomy can be safely performed in patients with cirrhosis if careful and appropriate management of bleeding and lymphorrhea from sites of dissection is ensured, is encouraging.


*Diagnostic and Therapeutic Endoscopy*, 1995, Vol. 1, pp. 147–152
Reprints available directly from the publisher
Photocopying permitted by license only

© 1995 Harwood Academic Publishers GmbH
Printed in Malaysia

### Laparoscopic Cholecystectomy for Cholelithiasis in Patients with Liver Cirrhosis

TETSURO ISHIKAWA, MICHIO SOWA, MASAYOSHI NAGAYAMA, and YUKIO NISHIGUCHI

*First Department of Surgery, Osaka City University Medical School 1-5-7, Asahi-machi, Abeno-ku, Osaka 545, Japan*

*(Received May 6, 1994; in final form May 20, 1994)*

We performed laparoscopic cholecystectomy for symptomatic cholelithiasis on four patients with cirrhosis of the liver, two of whom had clinical portal hypertension and splenomegaly. Preoperative examination disclosed hypersplenism in one patient, while mild thrombocytopenia and decreased prothrombin concentration were noted in three patients. However, no remarkable bleeding tendency was recognized clinically in any of the patients. Preoperatively, by Child-Pugh's criteria, three patients had class B disease and one class A disease. Intraoperatively, remarkable inflammatory change or fibrotic change of the gallbladder wall and Calot's triangle was observed in two cases, and collateral veins and lymphangial congestion were observed in all four cases. In the first case, extreme bleeding and lymphorrhea from dissected sites were observed, and a 1.5 unit of transfusion of whole blood was required during operation. Postoperatively, increase in ascites which was controlled with diuretics was recognized in one case. However, the postoperative course was uneventful in all cases, and no serious complications were recognized.

That laparoscopic cholecystectomy can be safely performed in patients with cirrhosis if careful and appropriate management of bleeding and lymphorrhea from sites of dissection is ensured, is encouraging.

**KEY WORDS:** liver cirrhosis, laparoscopic cholecystectomy, cholelithiasis, indications

### INTRODUCTION

Laparoscopic cholecystectomy has rapidly become the preferred method of managing symptomatic cholelithiasis. Absolute and relative contraindications to laparoscopic cholecystectomy are becoming fewer as surgeons gain experience and confidence in using the new equipment and techniques required for this procedure (1–3).

However, liver cirrhosis is still usually considered a relative or absolute contraindication to laparoscopic cholecystectomy, and only a few reports have been made of the performance of laparoscopic cholecystectomy in patients with liver cirrhosis. The indications for laparoscopic cholecystectomy in cirrhotic patients remain unclear and controversial (1,3,4).

For patients with cholelithiasis, our philosophy from the inception of laparoscopic cholecystectomy at our clinic has been to attempt to use laparoscopic procedures for all patients undergoing cholecystectomy. We report here the findings of four consecutive patients with liver cirrhosis who underwent successful laparoscopic cholecystectomy, and make some suggestions regarding effective intraoperative management of these patients.

### PATIENTS AND METHODS

The findings obtained for four consecutive patients with symptomatic cholelithiasis and cirrhosis of the liver who underwent laparoscopic cholecystectomy in our institute, were retrospectively reviewed. All four patients had been diagnosed with cirrhosis based on the results of pathologic examination. Clinical history, diagnosis, and preoperative data are listed for each patient in Table 1. Ultrasonography, computed tomography (CT) and drip infusion cholangiography (DIC) or endoscopic retrograde cholangiography (ERC) studies were performed preoperatively. Preoperative examination including blood cell counts, liver function tests including serum levels of total protein, albumin, aspartate aminotransferase, alanine aminotransferase, total bilirubin, alkaline phosphatase, and coagulation and fibrinolytic factors were performed

Address for correspondence: Tetsuro Ishikawa, M.D., 1st. Dept. of Surgery, Osaka City, University Medical School, 1-5-7 Asahi-machi, Abeno-ku Osaka 545 Japan.

**Table 1** Preoperative clinical features of patients with liver cirrhosis who underwent laparaoscopic cholecystectomy

| | *Case 1* | *Case 2* | *Case 3* | *Case 4* |
|---|---|---|---|---|
| Age, Sex | 57, female | 37, male | 57, female | 71, female |
| Clinical diagnosis | gallstone acute cholecystitis* | gallstone chronic cholecystitis | gallstone acute cholecystitis* | gallstone chronic cholecystitis |
| Type of liver cirrhosis | postnecrotic (HCV positive) | alcoholic (HBV positive) | postnecrotic (HCV positive) | postnecrotic (HCV positive) |
| Previous abdominal surgery | none | none | appendectomy | rt-nephrectomy |
| Preoperative associated conditions | EV, splenomegaly, DM | peptic ulcer | EV, splenomegaly, DM, HT | HT |
| Ascites controlled | controlled | controlled | none | |
| Chiled-Pugh classification | B | B | B | A |

*, treated with antibiotics.

EV, esophageal varices; DM, diabetes mellitus; HT, Hypertension.

for all four patients. Child-Pugh's classification (5) was used to grade the severity of liver dysfunction. Intraoperative findings including surgical procedure used, operating time, transfusion requirements, and operative complications were also recorded. Postoperative findings including duration of fever and use of analgesics, time until normal diet, and changes in liver function tests were also recorded.

### RESULTS

### Patients

Of 145 consecutive patients who underwent laparoscopic cholecystectomy between March 1992 and December 1993, 4 had cirrhosis of the liver (Figs. 1 and 2). They included 1 male and 3 females, between 37 and 71 years of age. Cases 1 and 3 manifested systemic signs of acute cholecystitis, such as high fever, right upper quadrant pains, and leucocytosis on each admission. These signs disappeared with antibiotic treatment before operation. Cases 2 and 3 suffered from right hypochondralgia and back pain. Three had postnecrotic hepatitis C viral infection and one had alcoholic cirrhosis combined with hepatitis B viral infection. Two had portal hypertension with esophageal varices and splenomegaly. The esophageal varices have been controlled with sclerotheraphy. Mild to moderate ascites were present in cases 1, 2, and 3, and controlled with diuretics (Table 1). All four patients had been demonstrated by ultrasonography to have gallstones. In case 3, DIC and ERC revealed nonfilling of the gallbladder. Preoperative examination (Table 2) disclosed thrombocytopenia in cases 1, 2, and 3, with platelet counts ranging between 49,000 and 96,000/mm$^3$, and leukocytopenia in cases 1 and 4 (2300 and 3100/mm$^3$, respectively). Liver function tests disclosed hypoalbuminemia (range, 2.5–3.2g/dl) and decreased hepaplastin time in all cases, and mild hyperbilirubinemia in cases 1, 3, and 4. Levels of indocyanine green excretion after 15 minutes (ICG $R_{15}$) were very high in cases 1 and 3, indicating severe hepatic dysfunction. Prothrombin and antithrombin III concentrations were below normal in cases 1, 2, and 3 (ranges, 69–73% and 52–56%, respectively), however, no bleeding tendency was clinically recognized in any patients. Preoperatively, by Child-Pugh's criteria, cases 1, 2, and 3 had class B disease, while case 4 had class A disease (Table 1).

### Surgical Procedure

All patients underwent general anesthesia and laparoscopy with a 0– or 30 degree forward viewing telescope, $CO_2$ insufflator, and electrocautery.

The first trocar was inserted using an open technique to avoid injury of collateral veins in the abdominal wall. The cystic duct and artery were exposed and ligated with titanium clips or absorbable monofilament ligatures. Intraoperative cholangiography was performed in routine fashion after identification of the cystic duct near its junction with the gallbladder. In cases 1 and 3, severe inflammatory and fibrotic changes around Calot's triangle were noted, and therefore dissection from the fundus to hilum of the gallbladder with frequent hemostasis was begun without dissection and isolation of the cystic duct. Following mobilization of the gall-

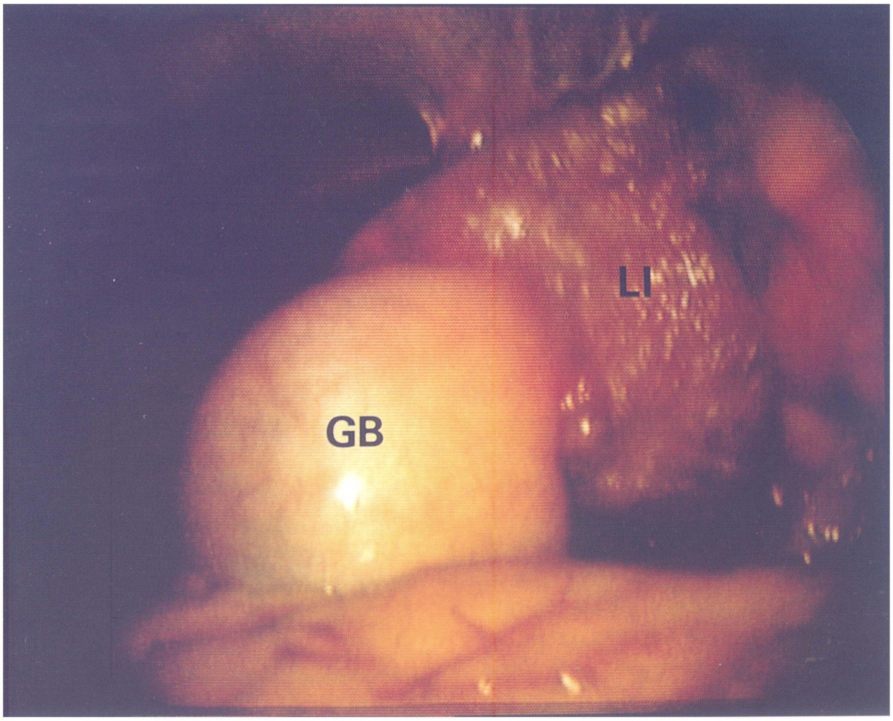

**Figure 1** Laparoscopic view in case 1. Liver atrophy and increase in thickness of the gallbladder wall are pronounced. And also, mild ascites are evident. GB, gallbladder; LI, liver.

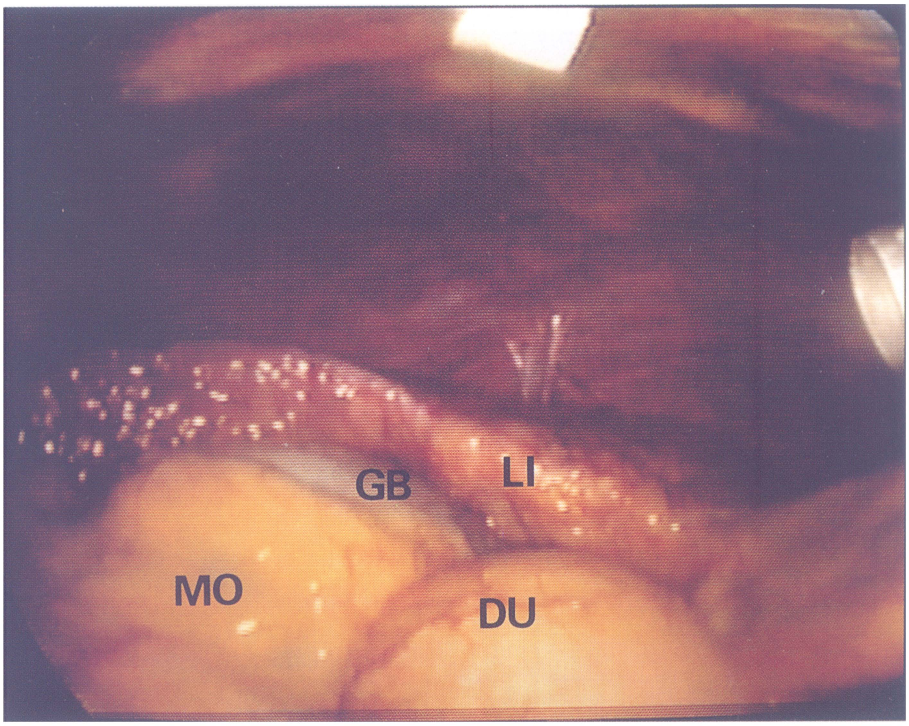

**Figure 2** Laparoscopic view in case 3. Gallbladder wall is hypertrophic and has fibrous adhesions to omentum and duodenum. GB, gallbladder; LI, liver; MO, major omentum; DU, duodenum.

**Table 2** Laboratory data on admission

| | *Case 1* | *Case 2* | *Case 3* | *Case 4* |
|---|---|---|---|---|
| RBC(× $10^4$/mm$^3$) | 320 | 370 | 390 | 360 |
| WBC(/mm$^3$) | 2300 | 5730 | 4300 | 3100 |
| Platelets(/mm$^3$) | 49,000 | 69,000 | 96,000 | 190,000 |
| TP(mg/dl) | 6.5 | 5.6 | 6.2 | 6.2 |
| ALB(g/dl) | 2.8 | 2.5 | 3.2 | 3.0 |
| AST(IU) | 36 | 88 | 50 | 124 |
| ALT(IU) | 37 | 97 | 73 | 68 |
| ALP(IU) | 286 | 193 | 212 | 268 |
| T-BIL(mg/dl) | 2.5 | 0.6 | 1.2 | 1.3 |
| ICG R15(%) | 48.9 | — | 25.5 | — |
| HPT(%) | 61 | 55 | 47 | 78 |
| PT%(%) | 70 | 69 | 73 | 80 |
| AT III (%) | 55 | 52 | 56 | 78 |

RBC, red blood cell; WBC, white blood cell; TP, total protein; ALB, albumin; AST, aspartate aminotransferase; ALT, alanine aminotransferase; T-BIL, total bilirubin; ALP, alkaline phosphatase; ICG R15, indocyanine green excretion after 15 minutes; HPT, hepaplastin time; PT%, concentration of prothrombin bladder, exposure of the cystic duct and artery became easier, and these structures were dissected (Fig. 3).

In cases 1 and 2, bleeding and lymphorrhea were controlled mainly with electrocautery, which resulted in extreme bleeding (1,100 and 600 ml, respectively) and lymphorrhea. Therefore, in cases 3 and 4, either clipping or ligation was frequently performed at sites of dessection. Fibrin glue was used to secure hemostasis at sites of dissection in cases 1 and 3, and a Penrose drain was placed.

Operating times (range, 105–370 minutes) and bleeding volumes (50–1,100 ml) are summarized in Table 3. A 1.5 unit whole blood transfusion was required in case 1 (Table 3).

### Postoperative Course and Outcome

In case 2, rebleeding from the abdominal wall on the first postoperative day was managed by transfusion of 1 unit of whole blood, and no surgical treatment was required. In case 3, moderate ascites developed, and was controlled with diuretics. There were no significant differences in results of liver function tests before and after operation (Table 4).

The postoperative courses were almost entirely uneventful, and no serious complications occurred in any of the 4 patients. Cases 1 and 4 were hospitalized for 6 and 7 days after surgery until skin sutures were removed. Discharge were delayed in two case, because of alcoholism in case 2, and of persistent ascites in case 3 (Table 5). Of the four patients, three are alive and without complaint between 7 and 11 months after surgery; in case 1, however, death due to rebleeding from esophageal varicies occurred 13 months after surgery.

**Table 3** Intraoperative course

| | *Case 1* | *Case 2* | *Case 3* | *Case 4* |
|---|---|---|---|---|
| Operating time(min) | 370 | 105 | 110 | 128 |
| Blood loss(ml) | 1100 | 600 | 130 | 50 |
| Blood transfusion(ml) | 600 | 0 | 0 | 0 |

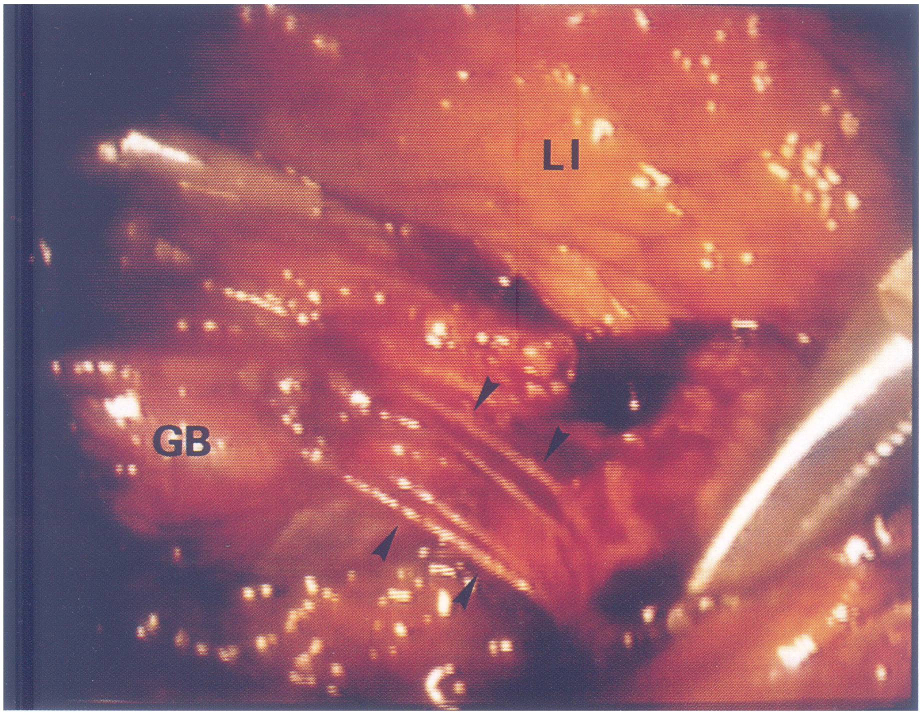

**Figure 3** Laparoscopic view in case 4. Hypervascularity of collateral veins and lymphoducts (➤) in Calot's triangle is observed. GB, gallbladder; LI, liver.

**Table 4** Changes in liver function tests and CRP values—peak values after operation compared with preoperative values

| | *Case 1* | *Case 2* | *Case 3* | *Case 4* |
|---|---|---|---|---|
| AST(IU) | 37 (36) | 59 (88) | 41 (50) | 82 (124) |
| ALT(IU) | 32 (37) | 43 (97) | 48 (73) | 95 (68) |
| ALP(IU) | 141 (186) | 106 (108) | 189 (286) | 152 (205) |
| T-BIL(mg/dl) | 2.5 (2.5) | 1.3 (0.6) | 0.9 (1.2) | 0.8 (1.3) |
| CRP(mg/dl) | 4.5 (0.4) | 5.1 (0.2) | 3.1 (0.3) | 2.6 (1.2) |

( ), Preoperative value; CRP, C-reactive protein.

### DISCUSSION

Conventional open cholecystectomy in patients with liver cirrhosis is a common but difficult clinical problem, since it is well established that this procedure carries a high risk of mortality and morbidity for these patients. Various authors (6–8) have demonstrated mortality rates ranging from 7.5% to 25.5%, and morbidity rates ranging from 12.2% to 23.6%. In addition, incidences of intraoperative transfusion ranging from 42.6% to 61.9% have been reported. Bloch and coworkers (8) suggested that operative intervention for symptomatic cholelithiasis is indicated for Child A or B patients before liver function deteriorates to class C status and before emergency intervention becomes necessary.

Laparoscopic cholecystectomy, which has the benefit of being a minimally invasive procedure (9,10) also has the possible disadvantage of poor control of significant intraoperative hemorrhage, since the same limitations in the use of surgical instruments and difficulties in placement of surgical packs or direct manual compression of bleeding sites under laparoscopic guidance exist. Given these considerations, many surgeons believe that cirrhosis is an absolute or relative contraindication to the performance of laparoscopic cholecystectomy. In addition, reports of the use of laparoscopic surgery in cirrhotic patients have appeared only occasionally (1,3,4)

From our experience, problems and measures taken to ensure safety in laparoscopic procedures include the following. At the commencement of surgery, the first trocar should be placed carefully so as not to injure the collateral veins. We have used an open technique and ligated or electrocauterized all sites of bleeding in all patients. Cirrhotic patients tend to have a gallbladder with a significant intrahepatic component due to atrophy of the right hepatic lobe, and, in addition, a hypertrophic left lobe which sometimes obscures the visual field of the telescope. Further difficulties sometimes arise when inflammatory changes such as scarring in the porta hepatis, hypertrophy of the gallbladder wall, and adhesions to the omentum and/or intestine are present. In difficult situations of this kind, an angled laparoscope is often useful for the identification of anatomical structures. Another problem in cirrhotic patients is hypervascularity, principally of collateral vein due to portal hypertension, of entire biliary lesion in cirrhotic patients. This is troublesome because of the difficulty in achieving local hemostasis. All four patients in our series had these findings, and therefore, meticulous dissection and hemostasis were required during all parts of the surgical procedure. Although electrocautery was used for most of the dissection in cases 1 and 2, hemo- and lymphostasis were insufficient, and extreme bleeding and lymphorrhea occurred from sites of dissection. We therefore recommend that clipping or ligation be performed at every site of dissection. In these procedures, however, longer operating times were taken (range, between 105 and 370 minutes), compared with that in patients without cirrhosis of the liver in our institute (80.1 ± 21.4 minutes).

We routinely performed intraoperative cholangiography (IOR) in order to confirm biliary duct anatomy and to exclude the presence of unsuspected biliary duct injury. IOR was simultaneously used to determine the point of dissection of the bile duct by measuring the distance between the fixing clip and common bile duct. We recommend that IOR be performed when the cystic duct is identified near its junction with the gallbladder; this may reduce overdissection into Calot's triangle and extensive bleeding.

No mortality or serious complications were observed, and the postoperative course was uneventful in all cases. No postoperative deterioration in liver function test values (serum AST, ALT, alkaline phosphatase or bilirubin) was noted in any of the four patients. Advantages of laparoscopic surgery also include shorter periods of postoperative fever and use of analgesics, as well as early ambulation.

In summary, despite the small number of patients studied and the short periods of follow-up, our initial results are encouraging enough to suggest that careful laparoscopic cholecystectomy for cholelithiasis in patients with cirrhosis who are Child-Pugh class A and B appears to be a safe and beneficial option.

**Table 5** Postoperative course

| | *Case 1* | *Case 2* | *Case 3* | *Case 4* |
|---|---|---|---|---|
| Duration of fever(≥ 37 °C) (days) | 2 | 2 | 2 | 1 |
| Duration of analgesic administration (days) | 1 | 1 | 1 | 0 |
| Time until normal diet (days) | 1 | 2 | 1 | 2 |
| Hospital stay (days) | 6 | 24 | 14 | 7 |
| Complications | none | bleeding* | ascites ** | none |

*, From abdominal wall; **, controlled with diuretics.

### REFERENCES

1. Frazee RC, Robert JW, Symmonds R, et al. What are the contraindications for laparoscopic cholecystectomy? Am J Surg, 1992;164:491–495
2. Morrell DG, Mullins JR, Harrison PB. Laparoscopic cholecystectomy during pregnancy in symptomatic patients. Surgery, 1992;112:856–859
3. Yerdel MA, Tsuge H, Mimura H, et al. Laparoscopic cholecystectomy in cirrhotic patients: expanding indications. Surg Laparosc Endosc, 1993;3:180–183
4. Soper NJ. Effect of nonbiliary problem on laparoscopic cholecystectomy. Am J Surg, 1993;165:522–526
5. Pugh NH, Murry-Lyon IM, Dawson JL, et al. Transection of the oesophagus for bleeding oesophageal varices. Brit J Surg, 1973; 60:646–649
6. Aranha GV, Sontag SJ, Greenlee HB. Cholecystectomy in cirrhotic patients: a formidable operation. Am J Surg, 1982;143:55–60
7. Garrison RN, Cryer HN, Howard DA, et al. Clarification of risk factors for abdominal operations in patients with hepatic cirrhosis. Ann Surg, 1984;199:648–655
8. Bloch RS, Allaben RD, Walt AJ. Cholecystectomy in patients with cirrhosis. Arch Surg, 1985;120:669–672
9. Gadacz TR. Comparison of open and laparoscopic cholecystectomy. Endoscopy, 1992;24:730–732
10. Ishikawa T, Nagayama M, Yamamoto Y, et al. A retrospective study of laparoscopic versus open cholecystectomy. Jpn J

